# Resistance to a CRISPR-based gene drive at an evolutionarily conserved site is revealed by mimicking genotype fixation

**DOI:** 10.1371/journal.pgen.1009740

**Published:** 2021-10-05

**Authors:** Silke Fuchs, William T. Garrood, Anna Beber, Andrew Hammond, Roberto Galizi, Matthew Gribble, Giulia Morselli, Tin-Yu J. Hui, Katie Willis, Nace Kranjc, Austin Burt, Andrea Crisanti, Tony Nolan

**Affiliations:** 1 Department of Life Sciences, Imperial College London, London, United Kingdom; 2 Department of Biology, University of Padua, Padua, Italy; 3 Department of Molecular Microbiology and Immunology, Bloomberg School of Public Health, Johns Hopkins University, Baltimore, United States of America; 4 Centre for Applied Entomology and Parasitology, School of Life Sciences, Keele University, Keele, Staffordshire, United Kingdom; 5 Department of Molecular Medicine, University of Padua, Padua, Italy; 6 Department of Vector Biology, Liverpool School of Tropical Medicine, Liverpool, United Kingdom; Fred Hutchinson Cancer Research Center, UNITED STATES

## Abstract

CRISPR-based homing gene drives can be designed to disrupt essential genes whilst biasing their own inheritance, leading to suppression of mosquito populations in the laboratory. This class of gene drives relies on CRISPR-Cas9 cleavage of a target sequence and copying (‘homing’) therein of the gene drive element from the homologous chromosome. However, target site mutations that are resistant to cleavage yet maintain the function of the essential gene are expected to be strongly selected for. Targeting functionally constrained regions where mutations are not easily tolerated should lower the probability of resistance. Evolutionary conservation at the sequence level is often a reliable indicator of functional constraint, though the actual level of underlying constraint between one conserved sequence and another can vary widely. Here we generated a novel adult lethal gene drive (ALGD) in the malaria vector *Anopheles gambiae*, targeting an ultra-conserved target site in a haplosufficient essential gene (AGAP029113) required during mosquito development, which fulfils many of the criteria for the target of a population suppression gene drive. We then designed a selection regime to experimentally assess the likelihood of generation and subsequent selection of gene drive resistant mutations at its target site. We simulated, in a caged population, a scenario where the gene drive was approaching fixation, where selection for resistance is expected to be strongest. Continuous sampling of the target locus revealed that a single, restorative, in-frame nucleotide substitution was selected. Our findings show that ultra-conservation alone need not be predictive of a site that is refractory to target site resistance. Our strategy to evaluate resistance *in vivo* could help to validate candidate gene drive targets for their resilience to resistance and help to improve predictions of the invasion dynamics of gene drives in field populations.

## Author summary

Gene drives have the potential to be applied as a novel control strategy of disease-transmitting mosquitoes, by spreading genetic traits that suppress or modify the target population. Many gene drive elements work by recognising and cutting a specific target sequence in the mosquito genome and copying themselves into that target sequence allowing the gene drive to increase in frequency in the population. Like other mosquito control interventions, efficacy will greatly depend on minimising the development of resistance to the gene drive mechanism—most likely via a change in the target sequence that prevents further cutting. One strategy to reduce resistance is to target sequences that are highly conserved, which implies that changes cannot easily be tolerated. We developed a strategy that simulates high selection pressure, under which resistance is most likely to emerge, and therefore provides a stringent test of its propensity to arise. Unlike previous results with another gene drive, we recovered a resistant allele within a few generations of gene drive exposure and at high frequency. Our results show that conserved sequences can vary hugely in ability to tolerate mutations and highlights the need to functionally validate future candidate gene drive target sites for their robustness to resistance.

## Introduction

CRISPR/Cas9 homing gene drives have shown much promise in providing an effective drive mechanism for novel genetic population modification and suppression strategies against insect vectors of major diseases, in particular the mosquitoes *Anopheles gambiae* and *Anopheles stephensi* [1–4]. In general, these gene drives comprise a genetic construct containing a source of ubiquitously expressed guide RNA (gRNA) and Cas9 endonuclease that is expressed in the germline and together they cut a very specific chromosomal target site. Homology Directed Repair (HDR) leads to copying over of the entire gene drive construct into the target site in the homologous chromosome, leading to homozygosity in the germline of carrier individuals. Therefore, unlike Mendelian inheritance, potentially all offspring will receive a copy of the transgene, regardless of which chromosome they inherit from the parent. Modelling has suggested that suppression of disease vector populations could be achieved by designing the gene drive to target and disrupt haplosufficient genes (i.e. genes where one functioning copy is sufficient to maintain normal function) that are essential in the soma for mosquito viability or fertility, and confining the homing reaction to the germline [5–7]. Under this scenario, individuals that contain one copy of the gene drive are ‘carriers’ that transmit the drive to a very high portion of the offspring, resulting in an increase in its frequency, which in turn places a strong genetic load—i.e reduction in fitness—on the population. If the load is high enough when the drive increases to its equilibrium frequency, population suppression or elimination can occur.

Selection of mutations at the target site, or pre-existing alleles in the population, however, can hinder this type of gene drive strategy and prevent further spreading of the drive element [8]. Mutations that prevent Cas9 cleavage at the intended cut site, whilst maintaining the function of the targeted gene, are referred to as ‘functional resistance” alleles. Such alleles can pre-exist in the target population or arise when the repair of the cleaved chromosome is mediated by non-homologous end joining (NHEJ) rather than HDR [8]. One approach to reduce the development of resistance to a gene drive is to target regions that are structurally or functionally constrained and therefore less likely to tolerate insertions or deletions (‘indels’) and substitutions that could lead to resistance.

As a proof of principle, this approach has been used to mitigate resistance in small cage/scale testing observed in previous applications that targeted female fertility genes [2]. By targeting a highly conserved 18bp sequence within the *A*. *gambiae doublesex* (*dsx*) gene, complete suppression of wild-type laboratory populations was achieved, while no alleles resistant to the gene drive were selected [1]. Although these results are promising for the use of population suppression gene drives against these malaria vectors, it is important to have a range of suitable target genes, allowing a more tailored approach to achieve the desired levels of suppression and avoidance of resistance. Moreover, the choice of suitable target gene will represent a trade-off between the magnitude of the load it imposes on a population when disrupted and its propensity to tolerate mutations that are resistant to the gene drive.

Our ability to identify novel genes containing gene drive targets that show high functional constraint has been greatly enhanced by genome resequencing efforts that have covered species across the *Anopheles* genus, spanning over 100 million years on an evolutionary timescale, as well as wild caught individuals of the closely related *Anopheles gambiae* and *Anopheles coluzzii* species [9–11]. A recent analysis of genomic data from 21 *Anopheles* species revealed over 8000 ultra-conserved sites (100% identity) of sufficient length (18bp; corresponding to the minimal sequence recognised by a guide RNA) to be targetable by homing-based gene drives [12]. It is to be expected that among this set of sequences there will be variation in their tolerance for changes to their underlying biological functions.

In this study, we focussed our analysis on ultra-conserved sites within putative haplosufficient essential genes as potential target sites for robust gene drives aimed at population suppression. Having generated an active gene drive targeting one of these essential genes, as proof of principle we subjected it to a maximal selection pressure to test its resilience to the selection of gene drive resistant alleles and its overall suitability for vector control.

## Results

### *AGAP029113* is a haplosufficient essential gene in *Anopheles gambiae*

A recent analysis of ultra-conserved elements (UCEs) from 21 different *Anopheles* species and search of functional annotations of genes containing UCEs by O’Loughlin et al [12] identified ultra-conserved targets potentially affecting female fertility or lethal recessive phenotype that could be suitable targets for vector control. Amongst the list of potential targets, the *AGAP029113* gene is of interest as it contains multiple ultra-conserved sites with a total of 140 invariant sites. Its apparent orthologue in *Drosophila melanogaster* is Smrter (*Smr)*, several null alleles of which lead to a recessive lethal phenotype, consistent with this gene being haplosufficient.

The *AGAP029113* gene is located on Chromosome 2L: 2895973–2931030 and was recently reassigned as a fusion of *AGAP004734* and *AGAP004732* (Vectorbase.org). It consists of 12 exons (S1 Fig) and contains domains putatively involved in interaction with the G-protein suppressor 2 pathway, DNA repair exonuclease subunit and DNA binding domains (Homeobox-like domain superfamily), suggesting a role in signalling, replication, recombination and DNA repair [13]. Its high level of conservation and expression in larvae and in female ovaries [14,15], which does not change with increasing adult age [16] suggests that this gene is required during mosquito development in both sexes [15].

We chose a target site within AGAP029113 that is ultra-conserved across Culicidae (Culicidae; last common ancestor ~ 150 million years ago) but not in *Drosophila melanogaster* genomes (Drosphilidae; 260 m.y.a.) (S1 Fig). Even within *An*. *gambiae* and *Anopheles coluzzii* populations (2784 wild *Anopheles* mosquitoes sampled across Africa), this particular target site showed only negligible variations of less than 0.6% frequency [17] (S1 Fig). This does not exclude the existence of further polymorphisms in wild populations but suggests that alteration of this sequence either naturally or by gene drive may have a strong fitness cost.

**Fig 1:**
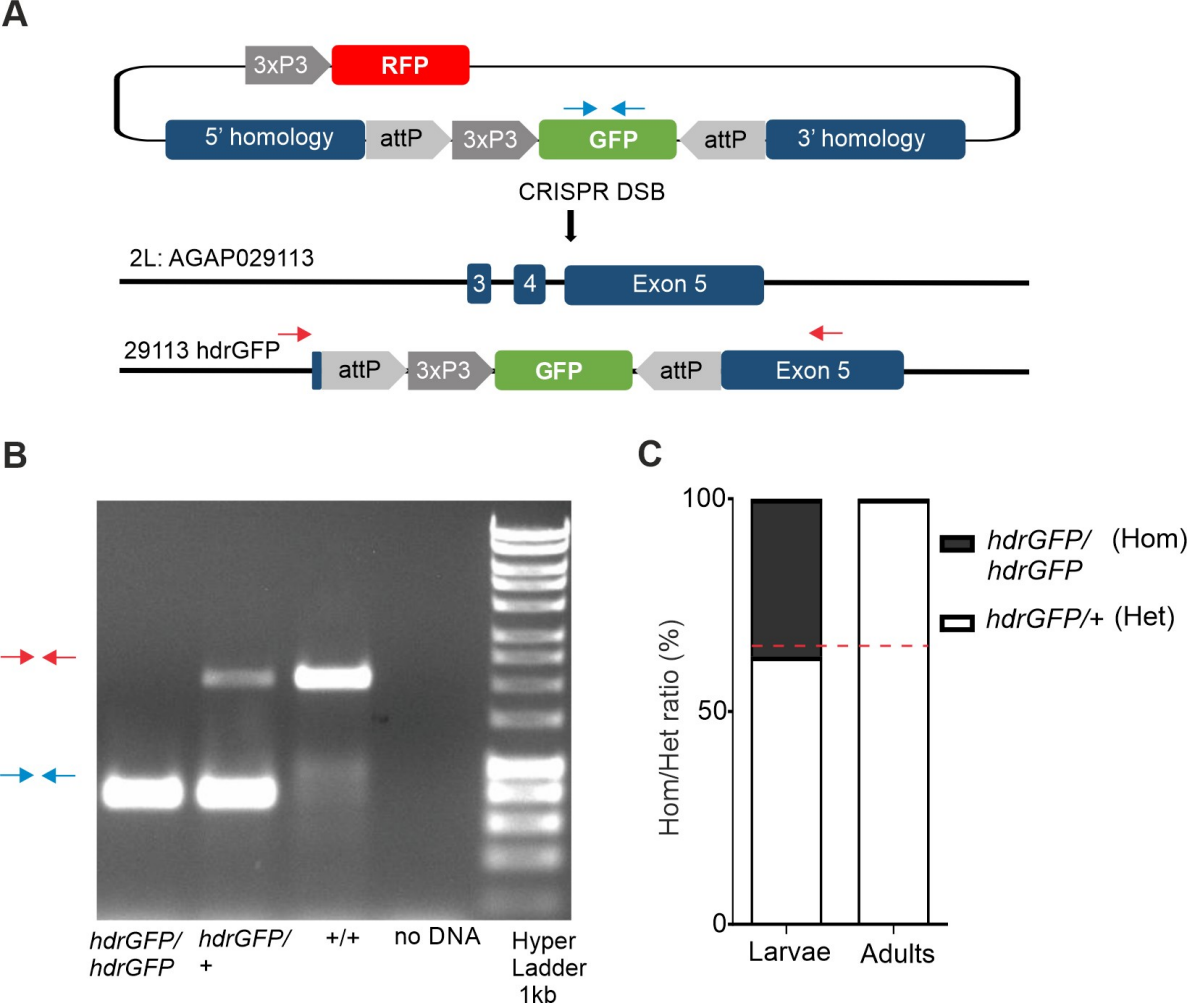
Gene disruption by HDR at a conserved site in exon 5 of *AGAP029113* causes recessive lethality. **(A)** Schematic representation of disruption of the *AGAP029113* gene. **(B)** PCR was used to confirm the targeted loci in WT individuals (red arrows) as well as those homozygous and heterozygous for the *29113*^*hdrGFP*^allele (blue arrow). **(C)** Heterozygous females and males were crossed with each other and the offspring was selected for GFP expression. Displayed is the percentage of homozygous (29113 ^hdrGFP/hdrGFP^) and heterozygous (29113 ^hdrGFP/+)^ L1 larvae (N = 60) and adults (N = 60) analysed by PCR as well as the expected homozygote vs heterozygote ratio according to Mendelian inheritance (red dashed line).

We then disrupted this candidate gene by inserting a GFP ‘docking’ cassette (‘*hdrGFP*’) into the target site within exon 5, which is expected to prevent the generation of a functional *AGAP029113* transcript and likely represents a null allele. The insertion of this cassette was performed by CRISPR-mediated HDR (Fig 1A), using previously described methodology [1,2]. After confirming the correct integration of the docking cassette (Fig 1B) we crossed heterozygous individuals with each other and scored their progeny. Among larvae we observed heterozygous (29113^hdrGFP/+^) and homozygous (29113^hdrGFP/hdrGFP^) individuals at the expected Mendelian ratio (Fig 1C). However, individuals homozygous for the null allele failed to emerge to adulthood. During general maintenance of the strain we noticed no obvious fitness effects in individuals heterozygous for the null allele, suggesting that this gene may represent a suitable haplosufficient essential gene as a target for a population suppression gene drive strategy, whereby ‘carriers’ with a gene drive disrupting a single copy of the target gene need to be competitive to be able to pass on the gene drive to their offspring.

### Phenotypic assessment of a gene drive targeting an essential gene

**Fig 2:**
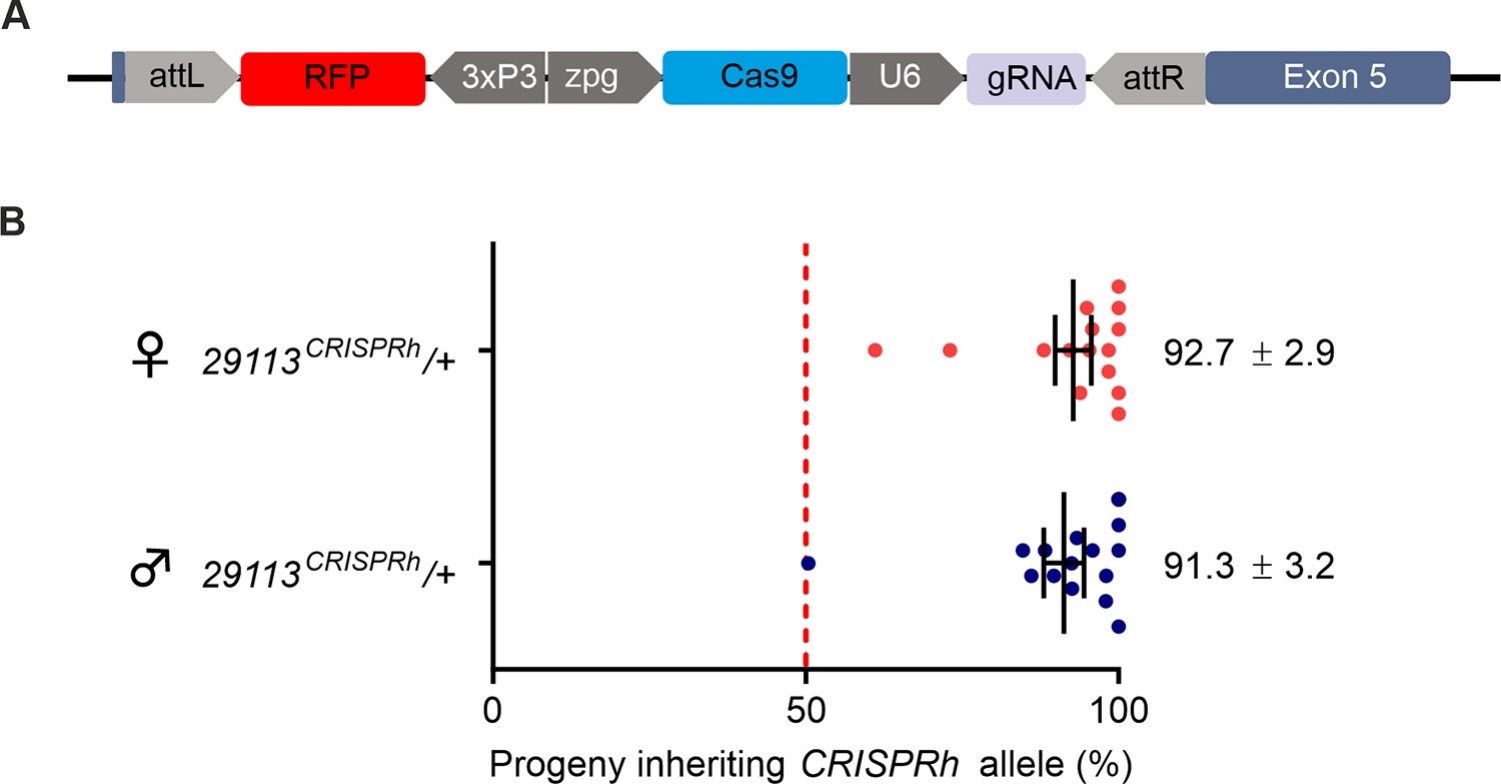
**Construct and homing rate of the *29113***^***CRISPRh***^ **allele (A)** Displayed is the CRISPR homing construct 29113^CRISPRh^ consisting of a *3xP3*::*RFP* marker, *Cas9* under the transcriptional control of the *zpg* promoter and a gRNA under the control of the ubiquitous *U6* PolII which replaced the GFP transcription unit in heterozygous 29113^hdrGFP/+^ lines by RCME. The gRNA cleaves at the homologous wild-type allele at the target site corresponding to the 29113^CRISPRh^ insertion. Repair of the cleaved chromosome through HDR leads to copying of the *CRISPR*^*h*^ allele and homing. **(B)** Male or female 29113 ^CRISPRh^/+ heterozygotes were mated to wild-type. High levels of homing of >90% was observed. Progeny from individual females were scored for the presence of the RFP linked to the 29113^CRISPRh^ construct and the average transmission rate indicated by vertical bar. A minimum of 15 females were analyzed for each cross. The average homing rate ± s.e.m. is also shown.

To assess the phenotype in the presence of a gene drive construct targeting this locus, an active Cas9/gRNA cassette (29113^CRISPRh^) was inserted into the locus as described previously [1,2]. The 29113^CRISPRh^ construct contained *Cas9* with expression controlled by the *zero population growth* (*zpg*) germline promoter [1], together with a dominant *RFP* marker gene to assist in tracking the inheritance of the gene drive (Fig 2A). This was placed in locus via recombinase mediated cassette exchange (RMCE), replacing the GFP cassette with the 29113^CRISPRh^ cassette. This gene drive construct showed high rates of biased inheritance from heterozygous (29113^CRISPRh/+^) female (92.7% ± 2.9) and male mosquitoes (91.3% ± 3.2) (Fig 2B).

**Fig 3:**
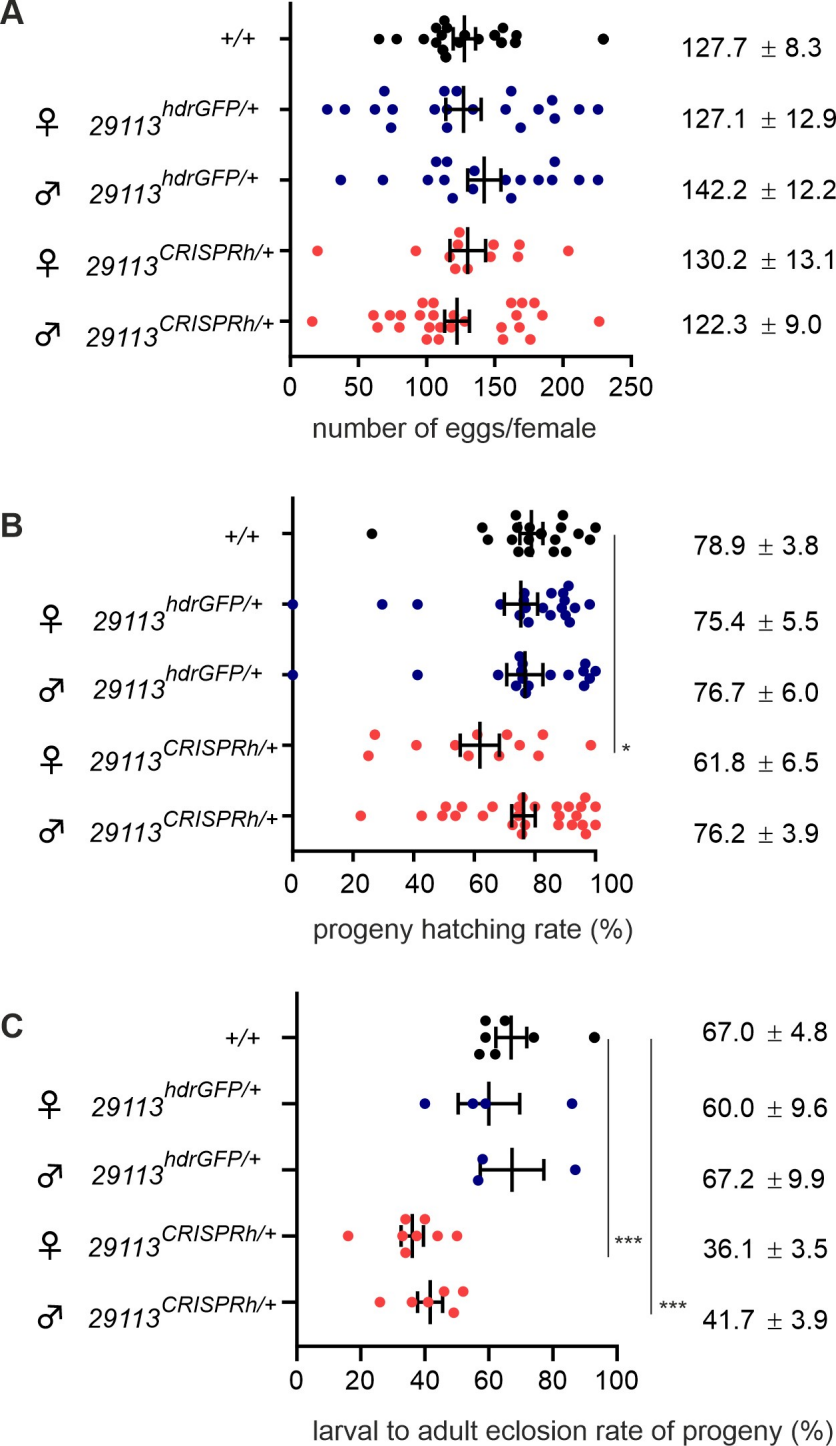
Phenotypic assessment of 29113^CRISPRh/+^ individuals crossed with wild-type. **(A)** Scatter plots displaying number of eggs laid by single females in crosses of 29113^*CRISPRh*^*/+* mosquitoes with wild-type individuals. Data were analyzed by quasi-Poisson GLM model. No significant differences were found between the wild-type and transgenic strains. **(B)** The hatching rate of these crosses is also displayed. Data were analyzed by quasi-binomial. No significant differences were found. **(C)** Larval to adult eclosion rate. Data were analyzed by a random effect binomial GLM model. * p<0.05, ** p<0.01, *** p<0.001.

Impaired fecundity and fertility of heterozygotes has been observed in other gene drive strains (1,2). We performed fecundity assays where heterozygous (29113^CRISPRh/+^) males and heterozygous (29113^CRISPRh/+^) females were crossed to wild-type G3 mosquitoes crossed repeatedly over multiple generations (to account for any parental effects as described in Hammond et al., 2021 [18]) and the number of eggs laid and hatched larvae was counted by individual oviposition of females. The wild-type G3 strain was used as control. There was no significant difference in the number of eggs across all 5 crosses (Fig 3A; quasi-Poisson GLM, F = 0.458, df1 = 4, df2 = 90, p = 0.766). However, the hatching rate of the progeny of 29113^CRISPRh/+^ females was reduced by 21.6% relative to wild-type controls (Fig 3B; z-test, p = 0.046). There was also increased mortality during larvae to adult development of about 37.8% and 46.2% among the offspring of heterozygous males and females, respectively (Fig 3C; z-test, 29113^CRISPRh/+^ males p = 3.33e-5; 29113^CRISPRh/+^ females p = 4.88e-8).

### Assessment of 29113^CRISPRh/+^ spread in a cage population experiment

Despite the fitness costs apparent with this gene drive in heterozygous carrier individuals, the extent of biased inheritance was still expected to cause the element to increase in frequency in a population [1,2]. To test this, we seeded 29113^*CRISPRh*/+^ individuals into a wild-type caged population (total initial population size of 400) at a starting frequency of 20%, in triplicate, and monitored the frequency of gene drive individuals over time. The same experiment was performed in parallel with the non-driving 29113 ^hdrGFP/+^ mosquitoes as control.

**Fig 4:**
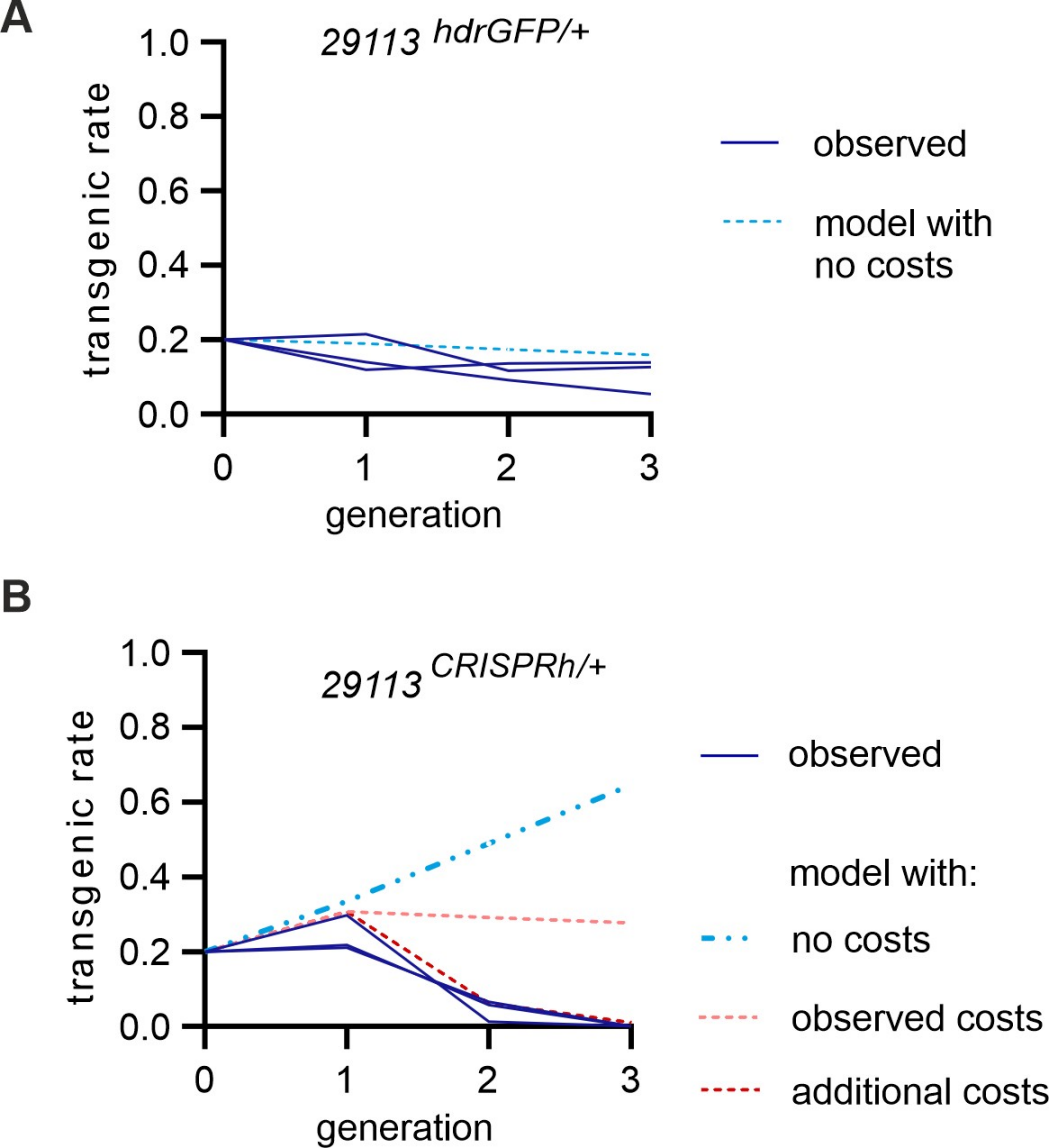
Frequency of individuals containing the 29113^CRISPRh^ drive allele over time in a population. Transgenics were introduced at a 20% frequency in a wild-type (G3) population. The transgenic rate was recorded in each subsequent generation. The experiments were performed in triplicates. **(A)** Proportion of individuals carrying the non-homing 29113^hdrGFP^ transgene (in blue). The dotted light blue line shows the expected transgenic rate, based on no fitness costs in heterozygotes **(B)** Proportion of 29113^CRISPRh/+^ individuals (in blue). Dotted lines show the deterministic prediction based on the observed homing rates for 29113^CRISPRh/+^ individuals assuming either no fitness costs in gene drive carriers (light blue hatched line), observed fitness costs (light red line) from the phenotypic assays (fecundity cost (1—relative hatching rate) (F) = 0.216 and somatic cost (1 –relative eclosion rate) (M) = 0.378, (F) = 0.462,) and additional somatic costs (M) = 0.9, (F) = 0.9, in dark red line) not measured in our experiments.

For releases with the control strain the frequency of individuals with the control allele decreased slowly over 3 generations to an average of 10.6%, consistent with the expectation for a non-driving null allele (Fig 4A). However, in the gene drive release cages, despite observing an initial increase in the frequency of gene drive carriers in the generation after release, this rapidly declined, to the extent that by 3 generations post-release the gene drive was lost from the population in all three replicates (Fig 4B). Rapid loss of the gene drive suggests that there is a fitness cost associated with the Cas9/gRNA expression from the gene drive allele that prevents its persistence. To understand possible fitness costs, we used a simple deterministic model of a single, randomly mating population with two life stages (juveniles and adults), two sexes and discrete non-overlapping generations, following the structure of Beaghton et al [19] and incorporated the heterozygote fitness costs identified from the phenotypic assays. They included a) no fitness costs (blue dashed line) b) fitness costs that were observed in the phenotypic assays (21% reduced hatching rate in 29113^CRISPRh/+^ females and reduced larvae to adult eclosion rate of 37.8% and 46.2% for 29113^CRISPRh/+^ males and females) (light red dashed line) and c) further fitness costs (90% for 29113^CRISPRh/+^ males and females) that were not measured and were chosen arbitrarily to signify a very high genetic load (dark red dashed line). Overall, the data fitted the model c) assuming high additional fitness costs associated with the gene drive construct. This could include, for example, fitness costs such as a prolonged larval developmental time or a reduced adult mating success, which we did not assess.

We also investigated the possibility of the generation and subsequent selection of a functional resistant allele to the gene drive that could have led to loss of the transgene. Such alleles can be formed when Cas9-mediated cuts are repaired by error-prone end joining DNA repair pathways producing small insertions or deletions or substitutions [8]. Our models showed that such resistant alleles would have had no impact on the transgenic rate over the first 5 generations of the population experiment (S2 Fig). This suggests that the dominant factor in determining the drive loss was the unexpected additional fitness costs associated with harbouring the drive element in heterozygosity, rather than the emergence and selection of resistance. Since these fitness effects were not observed in individuals heterozygous for the simple null allele, it suggests they are related to expression of the Cas9 nuclease, which may or may not be ameliorated in the future by changes to the design of this drive construct and its expression. Nonetheless, an important question is the resilience of this target site, and indeed any target site, to resistance in the face of a suppression gene drive.

### Non-drive alleles generated at the target site by Cas9 nuclease activity

**Fig 5:**
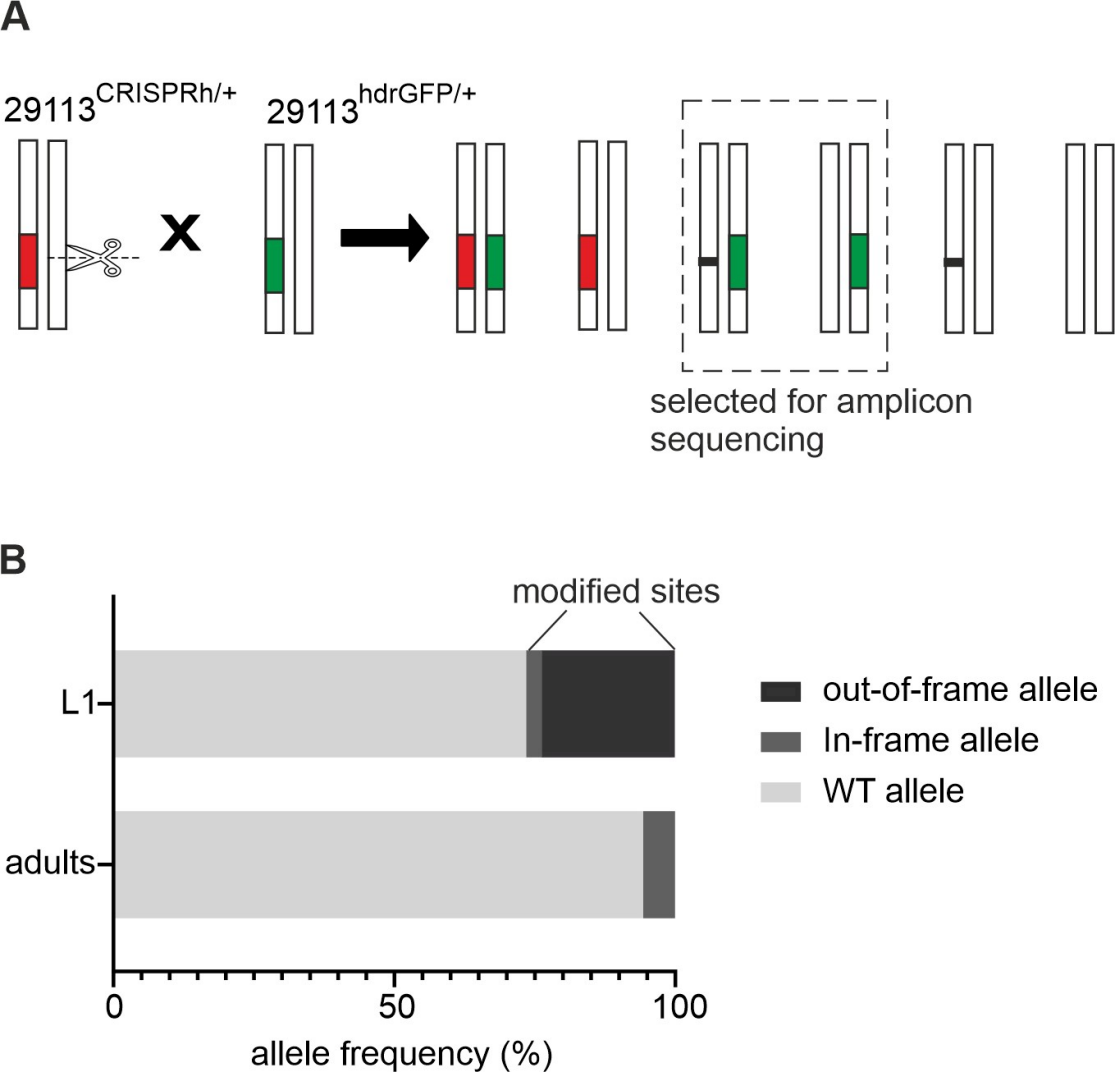
Resistance analysis of an ultra-conserved target site in *AGAP0029113*. **(A)** Crossing scheme devised to allow analysis of alleles that were exposed to CRISPR/Cas9, but which were not converted to the 29113^CRISPRh^ construct. The crossing of heterozygous 29113^CRISPRh/+^ individuals to 29113^hdrGFP/+^ individuals, and subsequent screening of the offspring for GFP+, provided mosquitoes that had an 29113^hdrGFP/+^ genotype, with the other allele being wild-type or containing indels generated by NHEJ repair. Red represents the 29113^CRISPRh^ allele (RFP+), whilst green shows the 29113^hdrGFP^ allele (GFP+) and black shows novel mutations. Progeny was screened for GFP+/RFP- (dashed rectangle). **(B)** Allele frequency (%) at the CRISPR/Cas9 cleavage site. L1: 7000 larvae, adults: 90 individuals.

To investigate the array of target site mutations that can be generated at the ultra-conserved site we devised a cross that enriched for non-drive alleles exposed to the nuclease from the gene drive (Fig 5A). In this assay the non-drive alleles are balanced against a marked (GFP+) null allele of the haplosufficient target gene. Consistent with an essential role for the *AGAP029113* gene between larval and adult transition, the distribution of in-frame and out-of-frame alleles was markedly different between the larval stage (where a complete null genotype is tolerated) and the adult stage (where it is not): pooled sequencing of the target site in L1 instar larvae revealed that 26.5% of non-drive alleles contained a target site mutation, of which 10.8% were in-frame (Fig 5B); sampling at the adult stage, only 5.7% of non-drive alleles contained mutations, of which 99.3% were in-frame. The persistence of in-frame alleles in the adult stage strongly suggests that these restore function, at least partially, to the essential target gene. If these mutations are also resistant to further cleavage by the gene drive then they would meet the criteria of being functionally resistant and thus be positively selected in the presence of the gene drive.

### Selection of 29113^CRISPRh^ resistant alleles by mimicking genotype fixation in a caged population

In order to investigate our assumption that these mutations could be strongly selected, we mimicked a scenario of genotype fixation (i.e., all mosquitoes have at least one copy of the gene drive), where selection pressure for functional resistance alleles is expected to be highest. We crossed individuals heterozygous for the gene drive construct, allowing all offspring to survive and reproduce for multiple generations. Given our previous estimate of gene drive transmission rate (93%), under this scenario the vast majority (87% (0.93^2^)) of progeny would be expected to be homozygous for the gene drive and so would die during larval development. Of the progeny that survive to form the following generation, the majority would be heterozygous and destined to produce predominantly non-viable offspring. Under these conditions a functional resistance allele will therefore have a high selective advantage since it will confer viability on any genotype that inherits it.

**Fig 6:**
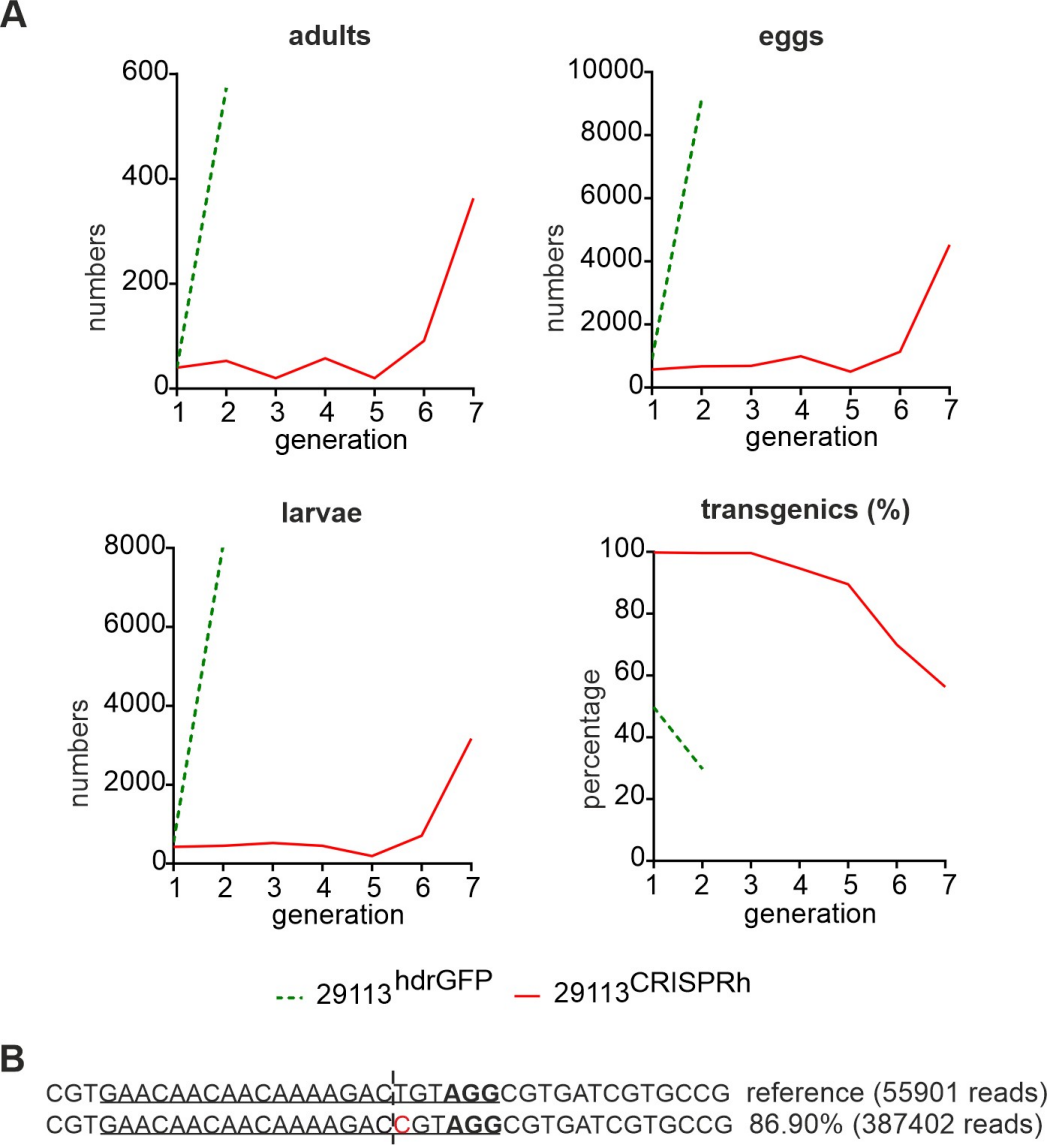
Reproductive output of a population when mimicking near genotype fixation of the 29113^CRISPRh^ allele. 20 heterozygous males and 20 heterozygous females for the 29113^CRISPRh^ allele were crossed with each other. The same cross was performed with 29113^hdrGFP/+^ individuals as control. **(A)** All adults, eggs and larvae were recorded. The resulting adults were used to set up the following generation. This procedure was continued until generation 7 (G7). The number of non-homing 29113^+/-^ adults was recorded as a comparison until G2, once the population had exceeded the carrying capacity of the experimental set up. **(B)** Indels and substitutions seen at the predicted cleavage site for 29113^CRISPRh^ were assessed in 220 adults (G7) after repeated crossing of 29113^CRISPRh/+^ individuals for 7 generations. The gRNA binding site is underlined, with the PAM highlighted in bold. The Base highlighted in red shows a substitution that was also identified in the resistance assay.

Over the first five generations of this experiment, the gene drive population (marked in red) was constantly suppressed (Fig 6A), producing very few eggs compared to the non-drive 29113^hdrGFP/+^ controls (in green), consistent with the majority of the offspring lacking a functional copy of the *AGAP029113* gene. However, by generations 6 and 7 we observed a rise in frequency of larvae as well as the number of adults and eggs produced by the gene drive cage. Together, these observations are consistent with a breakthrough of functional resistance alleles that are refractory to gene drive invasion and restore the carrying capacity of the population. Indeed, sequencing of adults from the G7 generation (Fig 6B) showed over 87% of reads contained the same in-frame mutation at the Cas9 target site. We also detected this single nucleotide substitution, which is silent, amongst other mutations recovered in larvae and adults in our previous screen (S3 Fig). Since this mutation was not found in the laboratory wild-type population (despite sequencing of 360 wild-type individuals and alignment of 35817 reads), the most likely explanation is that it is a *de novo* mutation caused by end-joining repair following nuclease activity of the gene drive. This specific mutation was selected and rapidly increased in frequency over generations likely due to a fitness advantage compared to other indels found in the single generation assay.

## Discussion

Any strategy, from antibiotics to insecticides, that aims to impose a fitness load on a population inevitably leads to strong selection for resistance. For gene drives, the easiest pathway for resistance to occur is for an allele to arise that codes for a functioning gene that lacks the endonuclease recognition site, thereby blocking the biased inheritance of the gene drive element. These alleles may pre-exist in the population, arise through spontaneous point mutation, or arise through error-prone (non-HDR) DNA repair pathways that introduce small mutations in the repair of the double stranded break generated by the gene drive.

We developed a CRISPR homing gene drive targeting an ultra-conserved site in the viability gene *AGAP0029113* and showed that this gene is essential for development of both male and female mosquitoes. However, the invasive potential of the gene drive construct was hindered by high fitness costs in the heterozygous 29113^CRISPRh^/+ individuals compared to the wild-type strain. Fitness costs in gene drive carriers are not unusual and have been reported for other gene drive strains [1,18,20,21]. A fitness cost, per se, in carriers will not prevent a gene drive increasing in frequency provided that the magnitude of its biased inheritance outweighs the fitness cost suffered in the carrier. However, in the case here, the fitness costs were severe enough to prevent the gene drive invading at all. One explanation for the fitness costs may be nuclease expression in the soma that converts the wild type allele to a null, thereby creating mosaic individuals with cells containing no functional copy of this essential gene. Mosaicism can derive from ‘leaky’ expression from the otherwise germline-restricted promoter or from parental deposition into the embryo. Indeed, the fraction of hatched eggs was reduced when the gene drive was present in the mother but not the father (Fig 3B), consistent with a small maternal contribution of Cas9 to the progeny. Though maternal effects of this kind are usually considered an inherent property of the germline promoter used to express Cas9, previous use of the *zpg* promoter has revealed a paternal [1], maternal (this study), or no parental effect at all [18] depending on its genomic site of integration and potentially its orientation with respect to nearby enhancers. Furthermore, we cannot exclude that the same level of mosaicism may lead to a stronger phenotypic effect at this target. An alternative explanation is that the target gene has a germline function in addition to its essential function in the soma, with the result that gene drive conversion to homozygosity in the germline leads to reduced fecundity. In our case it is difficult to disentangle such effects from general fitness effects since the competing hypothesis of somatic mosaicism could similarly lead to reduced fecundity.

Potentially such effects are more problematic for essential genes that are required during development in both sexes, compared to female fertility genes, since in the latter case heterozygous carrier males are unaffected. This is important for the phenotypic assessment of the suitability of future gene drive candidate genes required for viability in males and females and more emphasis should be placed on the restriction of gene drive activity to the germline. To look at the potential for gene drive resistance to arise at our chosen ultra-conserved target site, we devised both a single generation resistance assay and a population experiment designed to mimic the highest selection pressure. In both scenarios we recovered the same single point mutation at the target site. Possibly, the resistant allele we isolated might carry a small fitness cost which would be selected against in natural mosquito populations, which is why it was not detected within variation data from the 2784 *An*. *gambiae*, *An*. *coluzzii*, *Anopheles arabiensis* and hybrid individuals, collected from 19 countries in Africa as part of Phase 3 of the *Anopheles gambiae* 1000 Genomes Project [17]. However, when faced with a gene drive, any small fitness cost is offset by the relative advantage the mutation confers against the gene drive allele. Therefore, even where genomic locations are ultra-conserved across various species and populations over a wide geographic area, their mutation may be tolerated under the selective pressure of gene drive. Further, different ultra-conserved sites could have very different functional constraints and selection pressure; studies have shown that even without any obvious functional constraints sequences can remain ultra-conserved between different species [22]. Consequently, how to assess the potential of resistance development at any given conserved target site is of fundamental importance when considering gene drives of this type.

As a general rule, for a population suppression gene drive, the largest load is imparted on a population when the target is a gene essential for female fertility or viability, yet a considerable, though reduced, level of suppression can be achieved when the gene produces a recessive lethal phenotype that manifests in both sexes [23]. Given that there may be a paucity of genes with functionally constrained target sites that are sufficiently robust to resistance it may be that the optimal target site choice will consider a balance between the suppressive load conferred by the gene drive and the intrinsic capability of its target site to tolerate resistant mutations.

Our mimicking of genotype fixation could be a useful experimental approach for swiftly assessing target site susceptibility, since it isolates resistant alleles faster than possible in population experiments of gene drive spread into wild-type populations—the selective advantage to a resistant allele over a wild type allele is highest when the likelihood of either allele finding themselves balanced against a gene drive allele is highest. A similar rationale applies in the resistance assay balancing the gene drive against a marked null allele [18,24] that we also employed here, however in this assay no measure is given of how strongly such potentially resistant alleles are selected or, indeed, of whether they are resistant to the gene drive nuclease. One feature of targeting a gene essential in early development, as we did here, is that it allows a much higher throughput of screening, because selection happens during larval rearing, where rearing capacity is much less limited, meaning that all surviving adults have been through the selection bottleneck. In contrast, for a gene drive that targets female fertility in the adult, most adults in this scenario will be homozygous for the gene drive and selection of a resistant allele only occurs in the small subset of females that have the opportunity to mate and contain at least one non-drive allele.

It will be essential to build on successes to date that have shown gene drives that can suppress populations robustly, without obvious selection for resistance, for example when targeting the female isoform of the sex determination gene *doublesex* (1). In that specific case the gene drive targets a conserved site at an intron-exon boundary and it may be that the sequence is important at the RNA level and thus has even less degeneracy. Considerations such as this, or choosing target sites where the cleavage site lies in non-degenerate codons, or the non-wobble positions of degenerate codons, may add additional levels of resilience to a gene drive. However, at scale, in a field setting and considering the vast population sizes of mosquitoes, it is likely that such drives will need to be augmented by targeting multiple sites in the same gene, in order to greatly reduce the likelihood of multi-resistant alleles arising on the same haplotype [25–29]. A similar effect can be achieved by releasing multiple gene drives that independently target separate loci [23,30]. Moreover, using a similar logic, it could be prudent to have gene drives targeting genes in a range of different, independent biological pathways. In all cases, it will be essential to prioritise target site choice according not just to its suitability in terms of desired phenotypic effect when targeted, but its resilience to the generation of resistant alleles. Our results here show a pathway for how testing of ultra-conserved sites might proceed in order to evaluate these determinants for future gene drive designs in vivo.

## Materials and methods

### Ethics statement

All animal work was conducted according to UK Home Office Regulations and approved under Home Office License PPL 70/8914.

## Generation of CRISPR and donor constructs

A CRISPR construct (p16510) containing a human-codon-optimized *Cas9* coding sequence (*hCas9*) under the control of the *zpg* promoter and U6::gRNA spacer cloning cassette was utilized as described previously and modified using Golden Gate cloning to generate a PolIII transcription unit containing the AGAP0*29113*-specific gRNA [1,2]. The donor plasmid was assembled by MultiSite Gateway cloning (Invitrogen) and contained a *GFP* transcription unit under the control of the *3xP3* promoter enclosed within two reversible ϕC31 *attP* recombination sequences flanked both 5′ and 3′ by 2 kb sequence amplified using primer 29113 ex5 B1 f (CAACCAAGTAGTTACTGTGCTC) and 29113 ex5 B4 r (GTCTTTTGTTGTTGTTCACGT) and primer 29113 ex5 B3 f (TGTAGGCCGTGATCGTGC) and 29113 ex5 B2 r (GCGACACCATACTCCGATG) of the exon 5 target site in *AGAP029113*. To generate the 29113^CRISPRh^ allele a gRNA spacer (GAACAACAACAAAAGACTGTAGG) bearing complete homology to the intended target sequence was inserted by Golden Gate cloning into a CRISPR construct (p17410) as described previously [1,2]. This vector contains a human codon-optimized Cas9 gene (hCas9) under control of the zpg promoter and a U6::gRNA cassette with a BsaI cloning site, as well as a visual marker (3xP3::RFP). This construct sequence is flanked by attB recombination sites, which will allow the recombinase-mediated in vivo cassette exchange for the homing allele.

### Generation of the 29113^hdrGFP^ docking line and 29113^CRISPRh^ line

In order to generate the docking line, we injected 253 eggs of the *Anopheles gambiae* G3 strain with the 29113gRNA-modified p16510 plasmid (300ng/μl) and donor plasmid (300ng/μl) and obtained 7 transients that led to 6 transgenic individuals. Site-specific Integration was confirmed by PCR using primers binding the docking construct, 5′GFP-R (TGAACAGCTCCTCGCCCTTG) and a primer binding the genome outside of the homology arms: DL_29113 Ex5_F2 (TTCCACCTCTCGCTCGTAGT) and sequencing of the flanking sites. For the recombinase-mediated cassette exchange reactions a mix containing the CRISPR plasmid (200 ng/μl) and 400 ng/μl vasa2::integrase helper plasmid [31] was injected into embryos of the 29113^hdrGFP^ docking lines. Progeny from the outcross of surviving G_0_ individuals to WT were screened for the presence of RFP and the absence of GFP that should be indicative of a successful cassette-exchange event.

### Assessment of homing rate of CRISPR construct

To assess the ability of this 29113^CRISPRh^ construct to home at super-Mendelian rates, individuals heterozygous (29113^CRISPRh/+^) for the construct were crossed with wild type individuals and the progeny scored for inheritance of the CRISPR construct (via the proxy of RFP) from individual lays (N = 15 per cross).

### Molecular characterisation of homozygotes and heterozygotes for the 29113^hdrGFP^ allele

Males and females heterozygote for the 29113^hdrGFP^ allele were crossed with each other and 60 L1-L2 larvae were analysed by Multiplex PCR using primer 3’-GFP-F (GCCCTGAGCAAAGACCCCAA), 5’-GFP R (TGAACAGCTCCTCGCCCTTG) and the primer DL_29113 Ex5_F2 (TTCCACCTCTCGCTCGTAGT) flanking the transgenic construct. The same PCR was repeated for 60 adult mosquitoes from the same cross.

### Fertility and fecundity data

The 29113^CRISPRh^ strain was maintained in 2 batches by crossing either transgenic males with wild type females and by crossing transgenic females with wildtype males. This was done to account for any parental effects caused by deposition of the Cas9. The 29113^hdrGFP^ strain was maintained by crossing transgenic females with wildtype males. G roups of a minimum of 40 virgin heterozygotes carrying the 29113^hdrGFP/+^ or 29113^CRISPRh/+^ genotype respectively were mated to an equal number of virgin wildtype mosquitoes for 5 days and the number of eggs and offspring was counted from individuals lays (N≥12). Females that failed to lay eggs and did not contain sperm in their spermathecal were excluded from the analysis. Raw data including list of test statistics and sample sizes, as well all raw experimental data points, are included in S1_Data as an Excel file.

### Larval to adult eclosion rate

Groups of a minimum of 40 virgin heterozygotes carrying the 29113^hdrGFP/+^ or 29113^CRISPRh/+^ genotype respectively were mated to an equal number of virgin wildtype mosquitoes for 5 days. 100 transgenic L1 larvae were selected and reared to adulthood for each experiment (N≥3).

### Population cage experiments with 20% starting frequency

First instar mosquito larvae heterozygous for the 29113^CRISPRh^ allele were mixed within 12 h of eclosion with age-matched WT larvae at a ratio of 1:5 in rearing trays at a density of 200 per tray (in ∼1 litre rearing water). The mixed population was used to seed three cages (36cm^3^) with 400 adult mosquitoes each. For three generations, each cage was fed after allowing 5 days for mating, and an egg bowl placed in the cage 48 h after a blood meal to allow overnight oviposition. A random sample of 450 eggs was selected. After allowing full eclosion, offspring were scored under fluorescence microscopy for the presence or absence of the RFP-linked *CRISPR*^*h*^ allele, then reared together in the same trays and then used to populate the next generation.

### Population model

To model the results of the population cage experiment, we use a simple deterministic model of a single, randomly mating population with two life stages (juveniles and adults), two sexes and discrete non-overlapping generations, following the structure of Beaghton et al [19], without considering parental deposition. We initially consider 3 alleles: wild-type (W), transgenic (T) and cleavage resistant (R). We model insertion of the transgene into a recessive gene required for survival to adulthood and assume all cleavage resistant alleles are non-functional, therefore T/T, T/R and R/R adults are inviable. For the 29113^CRISPRh^ transgene, cleavage of the W allele occurs in W/T individuals, followed by either homology directed repair, converting W to T, or non-homologous end joining, converting W to R. Cleavage and end joining rates were calculated from the proportion of progeny inheriting the 29113^*CRISPRh*^ allele (*d*) (F) = 0.927, (M) = 0.913 (Fig 3B) and the probability that non-homed alleles are R (*u*) = 0.266 (based on % of exposed but unmodified alleles from Fig 5B) where *e* = 2*d*−1, cleavage = *e*+(1−*e*)*u*, (F) = 0.893, (M) = 0.872 and end joining (given cleavage) = $\frac{(1-e)u}{e+(1-e)u}$, (F) = 0.043, (M) = 0.053. To model functional resistance, we extend the model to include a fourth allele (r), which is both cleavage resistant and fully functional. Here we allow a proportion (*p*) of non-homologous end joining products to be functional (r) and 1−*p* to be non-functional (R). To incorporate the heterozygote fitness costs identified from the phenotypic assays we consider female heterozygotes carrying the transgene to lay fewer viable eggs compared to wildtype: fecundity cost (1—relative hatching rate) (F) = 0.216 (Fig 3B), and male and female heterozygotes to have reduced survival to adulthood: somatic cost (1 –relative eclosion rate) (M) = 0.378, (F) = 0.462, (Fig 3C). We also consider the case where heterozygotes carrying the transgene have additional somatic costs of other unknown factors: somatic cost (M) = 0.9, (F) = 0.9. In both cases W/R and W/r individuals are assumed to be fully fit. When modelling the 29113^hdrGFP^ line we assumed mendelian inheritance of the transgene and no heterozygous fitness costs. Following the experimental protocols, the frequency of transgenics was calculated at the juvenile stage, except for generation G_0_ which reflects that of the starting adult population. Code for running all the simulations is available on Github and linked to this article.

### Small population cage experiment mimicking fixation of the drive

20 male and 20 virgin female adults heterozygous for the 29113^*CRISPRh*^ allele were placed in a small cage (25cm^3^). Females were blood-fed after 5 days and an egg bowl was placed in the cage 48h post blood meal. After allowing full eclosion the number of eggs and RFP+ and RFP- larvae were recorded. All offspring were reared to adulthood and crossed with each other. This was continued for 7 generations. The same procedure was repeated for adults heterozygous for the 29113^hdrGFP^ allele for 2 generations. Offspring was screened for GFP+.

### Resistance assay

400 males heterozygous for the 29113^CRISPRh^ allele (RFP+) were crossed to 400 females heterozygous for the 29113^hdrGFP^ allele (GFP+). To collect L1 for DNA extractions, 7000 individuals were screened for GFP using COPAS. Larvae that were GFP+ only or RFP/GFP+ (RFP+ only and larvae with no fluorescent marker were discarded) were pooled and frozen in ethanol at -20°C. To collect adults for DNA extraction, large number of larvae were needed (as the majority would die before reaching adulthood). 10,000 L1 larvae were screened for GFP+, and the sorted larvae were split into 20 trays of 500 individuals. A single tray was then reassessed for frequency of GFP+ and RFP/GFP+. This tray was then rescreened at L4 stage for frequency of GFP+, RFP/GFP+ and the rate of larval mortality. Surviving larvae were collected from all 20 trays and screened manually for GFP+. These were allowed to emerge as adults before collection and storage at -20°C for later DNA extraction.

### PCR of target site and deep sequencing analysis preparation

Pooled DNA extractions (minimum 90 adult mosquitoes or 3500 L1 larvae) were performed using the Wizard Genomic DNA Purification kit (Promega). A 349 bp locus containing the predicted on-target cleavage site was amplified with primers containing Illumina Nextera Transposase adapters (underlined), 29113-F (TCGTCGGCAGCGTCAGATGTGTATAAGAGACAGCTGCTTGCGGGAACACTGTTAG) and 29113-R (GTCTCGTGGGCTCGGAGATGTGTATAAGAGACAGTGTTAAGGAAGTCCCGACAGCG). PCR reactions with KAPA HiFi HotStart ReadyMix PCR Kit (Kapa Biosystems) were setup with 80ng genomic DNA. These PCR reactions, library preparation and deep sequencing were performed as previously described [1].

### Deep sequencing analysis

CRISPResso software v2.0.29 [32] was utilised for analysis of amplicon sequencing around the on-target site, with the parameter–q 30. Reads containing indels and substitutions 1bp either side of the predicted cleavage site were tallied as modified. Frameshift analysis was performed within CRISPResso2 using parameter–c to input the coding sequence.

### Statistical analysis

To compare the number of eggs laid by different strains, a quasi-Poisson generalised linear model (GLM) was fitted to the egg counts, with stains (5 levels, or categories) being the main effect. Quasi-binomial was used to explore the difference in the hatching rates across. The quasi family was adopted as the data exhibits overdispersion. A mixed-effect binomial GLM was run to study the eclosion rates for the various strains and across generations. A random effect was included for experiments with replicates.

## Supporting information

S1 Fig Target site selection in *AGAP029113*. **(A)** Schematic representation of the 12 exons of AGAP029113 and location of the target site in exon 5 (white arrowhead). **(B)** Nucleotide alignment of exon 4 and 5 sections of AGAP029113 (Chromosome 2L: 2921441–2921488) belonging to *Anopheles gambiae/coluzzii* (AgamP3, AgamS1, AgamM1), *An*. *merus* (AmerM1), *An*. *arabiensis* (AaraD1), *Anopheles quadriannulatus* (AquaS1), *An*. *melas* (AmelC1), *Anopheles christyi* (AchrA1), *Anophles epiroticus* (AepiE1), *Anopheles farauti* (AfarF1), *Anopheles dirus* (AdirW1), *Anopheles darlingi* (AdarC2), *Anopheles albimanus* (AalbS1), *Anopheles atroparvus* (AatrE1), *Anopheles minimus* (AminM1), *Anopheles culicifacies* (AculA1), *Anopheles funestus* (AfunF1), *Anopheles stephensi* (AsteS1, AsteI2), *Anopheles maculatus* (AmacM1), *Anopheles sinensis* (AsinS1), *Ae*. *Albopictus* (Aealb), *Aedes aegypti* (Aeaeg), *Culex quinquefasciatus* (Cqui) and *Drosophila melanogaster* (Dmel). The CRISPR gRNA target site is underlined in red and the CRISPR-Cas9 cut site is indicated by a black arrowhead. The PAM sequence is indicated in bold letters. **(C)** Ag1000g phase 3 SNP data and frequency at the AGAP029113 target site sampled from 2784 wild *Anopheles* mosquitoes across Africa and 297 parents and progeny from 15 lab crosses. *Anopheles gambiae* (Agam), *Anopheles coluzzii* (Acol).
(TIF)

S2 Fig Deterministic prediction of the proportion of 29113^CRISPRh/+^ transgenics under observed fitness costs and varying probability of generation of a functional resistance (r) allele by NHEJ in the germline. (TIF)

S3 Fig Indels and substitutions seen at the predicted cleavage site for 29113CRISPRh in the resistance assay in larvae and adults. Crossing of heterozygous 29113^CRISPRh/+^ individuals to 29113^hdrGFP/+^ individuals, and subsequent screening of the offspring for GFP+, provided mosquitoes with a deficient 29113 allele, with the other allele being wild-type or containing indels generated by NHEJ repair. **(A)** L1 larvae (7000). **(B)** Adults (90). The gRNA binding site is underlined, with the PAM highlighted in bold. Horizontal dashes represent deletions whilst vertical dashes show the predicted cleavage site. Bases highlighted in red show substitutions, while bases in a red box represent insertions.
(TIF)

S1 Data Raw Data for Figs 1–6, S2 and S3. Includes all raw data points, lists of test statistics and sample sizes.
(XLSX)

## References

[1] Kyrou K, Hammond AM, Galizi R, Kranjc N, Burt A, Beaghton AK, et al. A CRISPR–Cas9 gene drive targeting *doublesex* causes complete population suppression in caged *Anopheles gambiae* mosquitoes. Nat Biotechnol. 2018 Nov 24;36(11):1062–6. doi: 10.1038/nbt.4245 30247490 PMC6871539

[2] Hammond A, Galizi R, Kyrou K, Simoni A, Siniscalchi C, Katsanos D, et al. A CRISPR-Cas9 gene drive system targeting female reproduction in the malaria mosquito vector *Anopheles gambiae*. Nat Biotechnol. 2016;34(1):78–83. doi: 10.1038/nbt.3439 26641531 PMC4913862

[3] Carballar-Lejarazú R, Ogaugwu C, Tushar T, Kelsey A, Pham TB, Murphy J, et al. Next-generation gene drive for population modification of the malaria vector mosquito, *Anopheles gambiae*. Proc Natl Acad Sci U S A. 2020;117(37):22805–14. doi: 10.1073/pnas.2010214117 32839345 PMC7502704

[4] Gantz VM, Jasinskiene N, Tatarenkova O, Fazekas A, Macias VM, Bier E, et al. Highly efficient Cas9-mediated gene drive for population modification of the malaria vector mosquito *Anopheles stephensi*. Proc Natl Acad Sci. 2015;112(49):E6736–43. doi: 10.1073/pnas.1521077112 26598698 PMC4679060

[5] Burt A. Site-specific selfish genes as tools for the control and genetic engineering of natural populations. Proc R Soc B Biol Sci. 2003;270(1518):921–8. doi: 10.1098/rspb.2002.2319 12803906 PMC1691325

[6] Eckhoff PA, Wenger EA, Godfray HCJ, Burt A. Impact of mosquito gene drive on malaria elimination in a computational model with explicit spatial and temporal dynamics. Proc Natl Acad Sci. 2017;114(2):E255–64. doi: 10.1073/pnas.1611064114 28028208 PMC5240713

[7] Godfray HCJ, North A, Burt A. How driving endonuclease genes can be used to combat pests and disease vectors. BMC Biol. 2017 Dec 11;15(1):81. doi: 10.1186/s12915-017-0420-4 28893259 PMC5594614

[8] Hammond AM, Kyrou K, Bruttini M, North A, Galizi R, Karlsson X, et al. The creation and selection of mutations resistant to a gene drive over multiple generations in the malaria mosquito. Barton NH, editor. PLOS Genet. 2017 Oct 4;13(10):e1007039. doi: 10.1371/journal.pgen.1007039 28976972 PMC5648257

[9] Neafsey DE, Waterhouse RM, Abai MR, Aganezov SS, Alekseyev MA, Allen JE, et al. Highly evolvable malaria vectors: The genomes of 16 *Anopheles* mosquitoes. Science (80-). 2015 Jan 2;347(6217):1258522. doi: 10.1126/science.1258522 25554792 PMC4380271

[10] Miles A, Harding NJ, Bottà G, Clarkson CS, Antão T, Kozak K, et al. Genetic diversity of the African malaria vector *Anopheles gambiae*. Nature. 2017 Dec 29;552(7683):96–100. doi: 10.1038/nature24995 29186111 PMC6026373

[11] Clarkson CS, Miles A, Harding NJ, Lucas ER, Battey CJ, Amaya-Romero JE, et al. Genome variation and population structure among 1142 mosquitoes of the African malaria vector species *Anopheles gambiae* and *Anopheles coluzzii*. Genome Res. 2020 Oct;30(10):1533–46. doi: 10.1101/gr.262790.120 32989001 PMC7605271

[12] O’Loughlin SM, Forster AJ, Fuchs S, Dottorini T, Nolan T, Crisanti A, et al. Ultra-conserved sequences in the genomes of highly diverse *Anopheles* mosquitoes, with implications for malaria vector control. G3 Genes|Genomes|Genetics. 2021 Mar 17; 10.1093/g3journal/jkab086 PMC8495744 33730159

[13] Marchler-Bauer A, Bo Y, Han L, He J, Lanczycki CJ, Lu S, et al. CDD/SPARCLE: functional classification of proteins via subfamily domain architectures. Nucleic Acids Res. 2017 Jan 4;45(D1):D200–3. doi: 10.1093/nar/gkw1129 27899674 PMC5210587

[14] Baker D, Nolan T, Fischer B, Pinder A. A comprehensive gene expression atlas of sex-and tissue-specificity in the malaria vector, *Anopheles gambiae*. BMC Genomics. 2011;12(1):296. doi: 10.1186/1471-2164-12-296 21649883 PMC3129592

[15] Marinotti O, Calvo E, Nguyen QK, Dissanayake S, Ribeiro JMC, James AA. Genome-wide analysis of gene expression in adult *Anopheles gambiae*. Insect Mol Biol. 2006 Feb;15(1):1–12. doi: 10.1111/j.1365-2583.2006.00610.x 16469063

[16] Cook PE, Sinkins SP. Transcriptional profiling of *Anopheles gambiae* mosquitoes for adult age estimation. Insect Mol Biol. 2010 Dec;19(6):745–51. doi: 10.1111/j.1365-2583.2010.01034.x 20695922 PMC2998705

[17] The *Anopheles gambiae* 1000 Genomes Consortium. Ag1000G phase 3 SNP data release. MalariaGEN. 2021.

[18] Hammond A, Karlsson X, Morianou I, Kyrou K, Beaghton A, Gribble M, et al. Regulating the expression of gene drives is key to increasing their invasive potential and the mitigation of resistance. PLoS Genet. 2021;17(1):1–21. doi: 10.1371/journal.pgen.1009321 33513149 PMC7886172

[19] Beaghton AK, Hammond A, Nolan T, Crisanti A, Burt A. Gene drive for population genetic control: non-functional resistance and parental effects. Proceedings Biol Sci. 2019 Nov 6;286(1914):20191586. doi: 10.1098/rspb.2019.1586 31662083 PMC6842858

[20] Kandul NP, Liu J, Bennett JB, Marshall JM, Akbari OS. A confinable home-and-rescue gene drive for population modification. Elife. 2021 Mar 5;10:1–25. doi: 10.7554/eLife.65939 33666174 PMC7968924

[21] Terradas G, Buchman AB, Bennett JB, Shriner I, Marshall JM, Akbari OS, et al. Inherently confinable split-drive systems in *Drosophila*. Nat Commun. 2021 Dec 5;12(1):1480. doi: 10.1038/s41467-021-21771-7 33674604 PMC7935863

[22] Díaz-Castillo C, Xia X-Q, Ranz JM. Evaluation of the Role of Functional Constraints on the Integrity of an Ultraconserved Region in the Genus *Drosophila*. Noor MAF, editor. PLoS Genet. 2012 Feb 2;8(2):e1002475. doi: 10.1371/journal.pgen.1002475 22319453 PMC3271063

[23] Deredec A, Burt A, Godfray HCJ. The Population Genetics of Using Homing Endonuclease Genes in Vector and Pest Management. Genetics. 2008 Aug 1;179(4):2013–26. doi: 10.1534/genetics.108.089037 18660532 PMC2516076

[24] KaramiNejadRanjbar M, Eckermann KN, Ahmed HMM, Sánchez C HM, Dippel S, Marshall JM, et al. Consequences of resistance evolution in a Cas9-based sex-conversion suppression gene drive for insect pest management. Proc Natl Acad Sci U S A. 2018;201713825. 10.1073/pnas.1713825115 PMC6004448 29844184

[25] Champer J, Liu J, Oh SY, Reeves R, Luthra A, Oakes N, et al. Reducing resistance allele formation in CRISPR gene drive. Proc Natl Acad Sci. 2018;115(21):5522–7. doi: 10.1073/pnas.1720354115 29735716 PMC6003519

[26] Oberhofer G, Ivy T, Hay BA. Cleave and Rescue, a novel selfish genetic element and general strategy for gene drive. Proc Natl Acad Sci. 2019 Mar 26;116(13):6250–9. doi: 10.1073/pnas.1816928116 30760597 PMC6442612

[27] Champer J, Yang E, Lee E, Liu J, Clark AG, Messer PW. A CRISPR homing gene drive targeting a haplolethal gene removes resistance alleles and successfully spreads through a cage population. Proc Natl Acad Sci. 2020 Sep 29;117(39):24377–83. doi: 10.1073/pnas.2004373117 32929034 PMC7533649

[28] Kandul NP, Liu J, Buchman A, Gantz VM, Bier E, Akbari OS. Assessment of a Split Homing Based Gene Drive for Efficient Knockout of Multiple Genes. G3 Genes|Genomes|Genetics. 2020 Feb 1;10(2):827–37. doi: 10.1534/g3.119.400985 31882406 PMC7003086

[29] Champer SE, Oh SY, Liu C, Wen Z, Clark AG, Messer PW, et al. Computational and experimental performance of CRISPR homing gene drive strategies with multiplexed gRNAs. Sci Adv. 2020 Mar 4;6(10):eaaz0525. doi: 10.1126/sciadv.aaz0525 32181354 PMC7056305

[30] Deredec A, Godfray HCJ, Burt A. Requirements for effective malaria control with homing endonuclease genes. Proc Natl Acad Sci. 2011;108(43):E874–80. doi: 10.1073/pnas.1110717108 21976487 PMC3203790

[31] Volohonsky G, Terenzi O, Soichot J, Naujoks DA, Nolan T, Windbichler N, et al. Tools for *Anopheles gambiae* Transgenesis. G3 Genes|Genomes|Genetics. 2015 Jun 10;5(6):1151–63. doi: 10.1534/g3.115.016808 25869647 PMC4478545

[32] Clement K, Rees H, Canver MC, Gehrke JM, Farouni R, Hsu JY, et al. CRISPResso2 provides accurate and rapid genome editing sequence analysis. Nat Biotechnol. 2019 Mar 26;37(3):224–6. doi: 10.1038/s41587-019-0032-3 30809026 PMC6533916

