# Supplementary figures and images for: Resistance to a CRISPR-based gene drive at an evolutionarily conserved site is revealed by mimicking genotype fixation
Source: PLoS Genet. 2021 Oct 5;17(10):e1009740. doi: 10.1371/journal.pgen.1009740 (PMC8519452; doi:10.1371/journal.pgen.1009740)

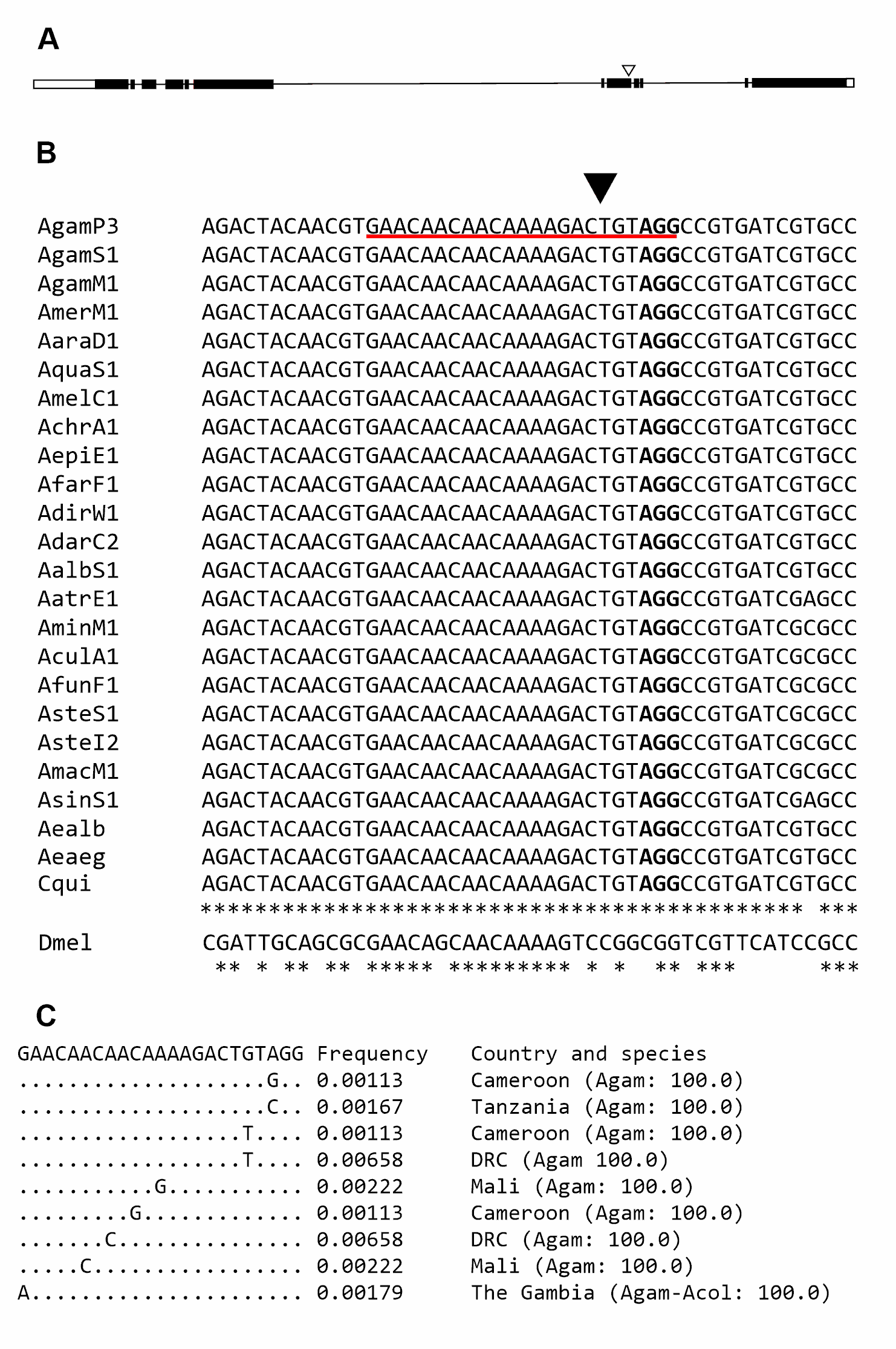

Supplement: S1 Fig — (A) Schematic representation of the 12 exons of AGAP029113 and location of the target site in exon 5 (white arrowhead). (B) Nucleotide alignment of exon 4 and 5 sections of AGAP029113 (Chromosome 2L: 2921441–2921488) belonging to Anopheles gambiae/coluzzii (AgamP3, AgamS1, AgamM1), An. merus (AmerM1), An. arabiensis (AaraD1), Anopheles quadriannulatus (AquaS1), An. melas (AmelC1), Anopheles christyi (AchrA1), Anophles epiroticus (AepiE1), Anopheles farauti (AfarF1), Anopheles dirus (AdirW1), Anopheles darlingi (AdarC2), Anopheles albimanus (AalbS1), Anopheles atroparvus (AatrE1), Anopheles minimus (AminM1), Anopheles culicifacies (AculA1), Anopheles funestus (AfunF1), Anopheles stephensi (AsteS1, AsteI2), Anopheles maculatus (AmacM1), Anopheles sinensis (AsinS1), Ae. Albopictus (Aealb), Aedes aegypti (Aeaeg), Culex quinquefasciatus (Cqui) and Drosophila melanogaster (Dmel). The CRISPR gRNA target site is underlined in red and the CRISPR-Cas9 cut site is indicated by a black arrowhead. The PAM sequence is indicated in bold letters. (C) Ag1000g phase 3 SNP data and frequency at the AGAP029113 target site sampled from 2784 wild Anopheles mosquitoes across Africa and 297 parents and progeny from 15 lab crosses. Anopheles gambiae (Agam), Anopheles coluzzii (Acol). (TIF) [file pgen.1009740.s001.tif]

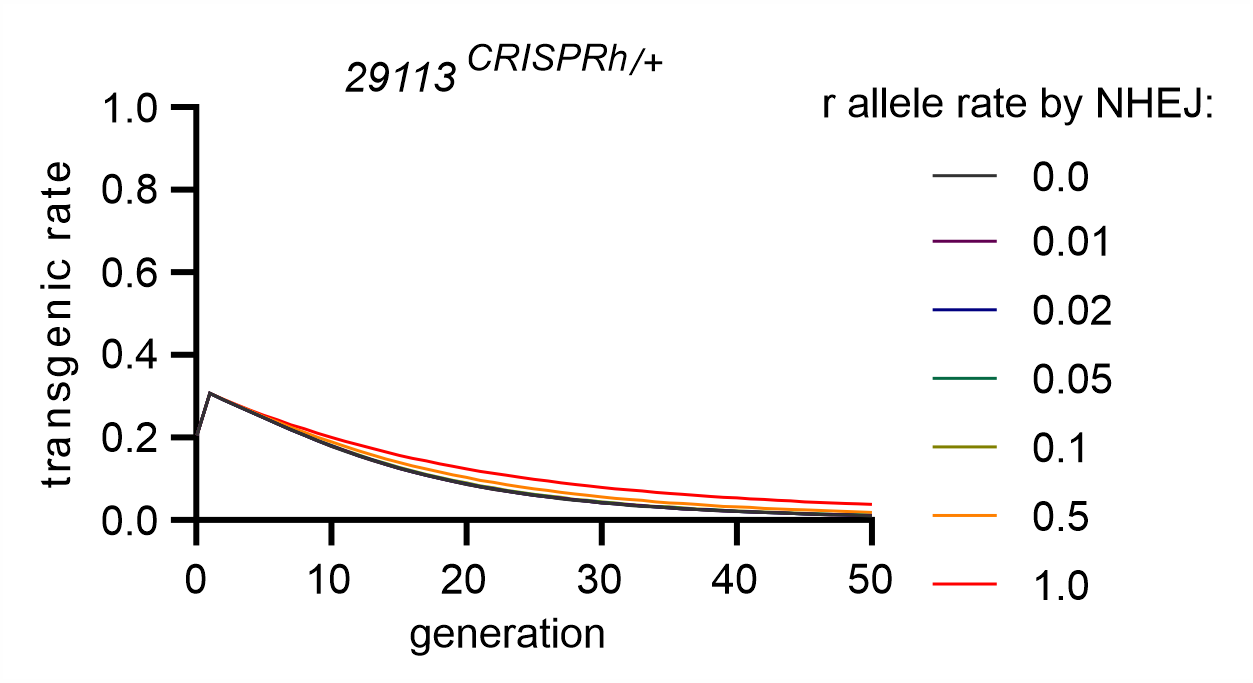

Supplement: S2 Fig — (TIF) [file pgen.1009740.s002.tif]

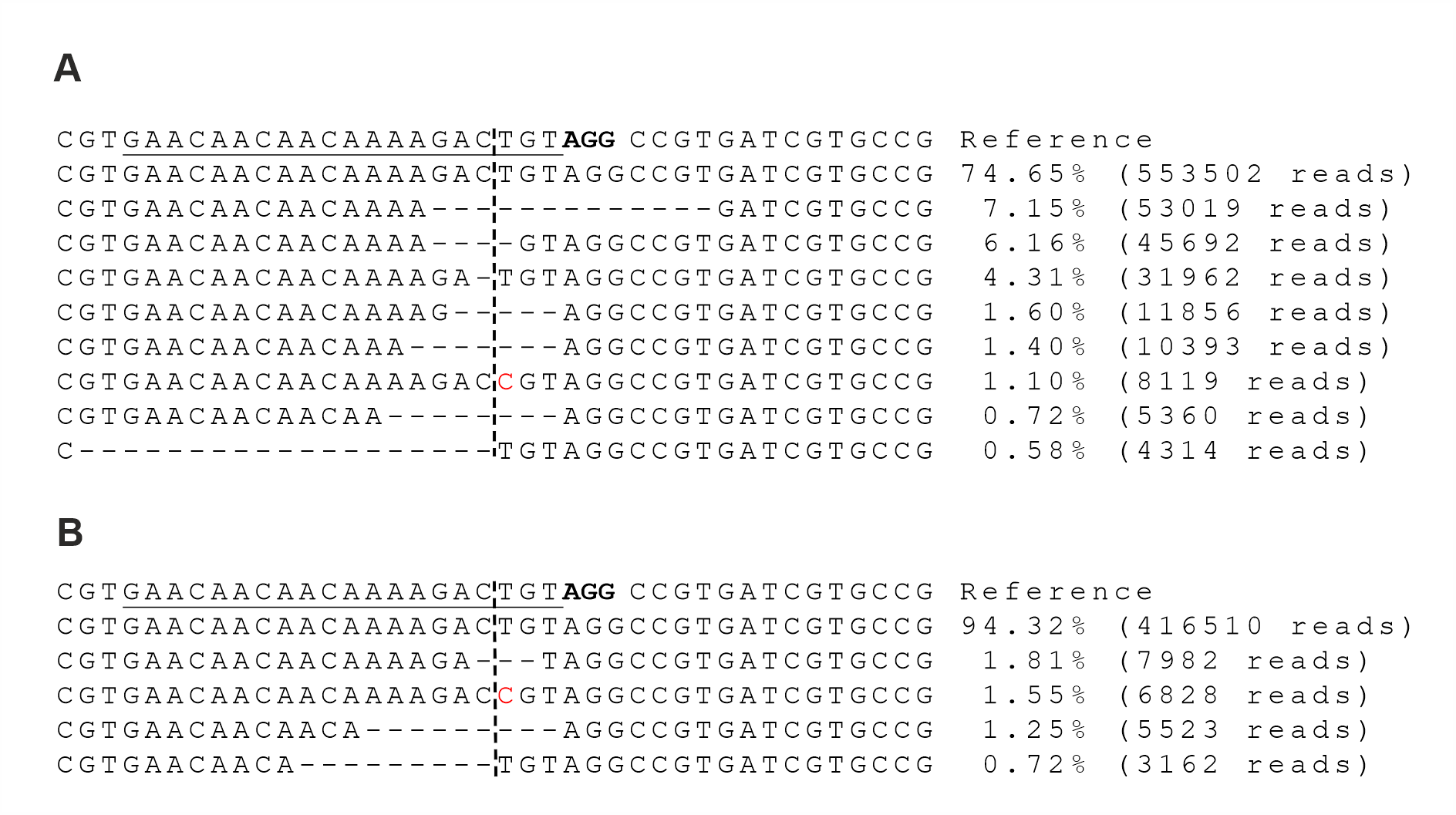

Supplement: S3 Fig — Crossing of heterozygous 29113CRISPRh/+ individuals to 29113hdrGFP/+ individuals, and subsequent screening of the offspring for GFP+, provided mosquitoes with a deficient 29113 allele, with the other allele being wild-type or containing indels generated by NHEJ repair. (A) L1 larvae (7000). (B) Adults (90). The gRNA binding site is underlined, with the PAM highlighted in bold. Horizontal dashes represent deletions whilst vertical dashes show the predicted cleavage site. Bases highlighted in red show substitutions, while bases in a red box represent insertions. (TIF) [file pgen.1009740.s003.tif]
